# Haplotype Association between Haptoglobin (Hp2) and Hp Promoter SNP (A-61C) May Explain Previous Controversy of Haptoglobin and Malaria Protection

**DOI:** 10.1371/journal.pone.0000362

**Published:** 2007-04-11

**Authors:** Sharon E. Cox, Conor Doherty, Sarah H. Atkinson, Chidi V. Nweneka, Anthony J.C. Fulford, Hala Ghattas, Kirk A. Rockett, Dominic P. Kwiatkowski, Andrew M. Prentice

**Affiliations:** 1 Medical Research Council (MRC) International Nutrition Group, London School of Hygiene and Tropical Medicine (LSHTM), London, United Kindgom; 2 Medical Research Council (MRC) Keneba, MRC Laboratories, The Gambia; 3 Wellcome Trust Centre for Human Genetics, Oxford, United Kingdom; DER Neurogenetics, National Institute of Neurological Disorders and Stroke, United States of America

## Abstract

**Background:**

Malaria is one of the strongest recent selective pressures on the human genome, as evidenced by the high levels of varying haemoglobinopathies in human populations–despite the increased risk of mortality in the homozygous states. Previously, functional polymorphisms of Hp, coded by the co-dominant alleles Hp1 and Hp2, have been variously associated with several infectious diseases, including malaria susceptibility.

**Methodology/Principal Findings:**

Risk of a clinical malarial episode over the course of a malarial transmission season was assessed using active surveillance in a cohort of Gambian children aged 10–72 months. We report for the first time that the major haplotype for the A-61C mutant allele in the promoter of haptoglobin (Hp)–an acute phase protein that clears haemoglobin released from haemolysis of red cells–is associated with protection from malarial infection in older children, (children aged ≥36 months, >500 parasites/ul and temperature >37.5°C; OR = 0.42; [95% CI 0.24–0.73] p = 0.002) (lr test for interaction, <36 vs ≥36 months, p = 0.014). Protection was also observed using two other definitions, including temperature >37.5°C, dipstick positive, plus clinical judgement of malaria blinded to dipstick result (all ages, OR = 0.48, [95% CI 0.30–0.78] p = 0.003; ≥36 months, OR = 0.31, [95% CI 0.15–0.62] p = 0.001). A similar level of protection was observed for the known protective genetic variant, sickle cell trait (HbAS).

**Conclusions/Significance:**

We propose that previous conflicting results between Hp phenotypes/genotypes and malaria susceptibility may be explained by differing prevalence of the A-61C SNP in the populations studied, which we found to be highly associated with the Hp2 allele. We report the -61C allele to be associated with decreased Hp protein levels (independent of Hp phenotype), confirming *in vitro* studies. Decreased Hp expression may lead to increased oxidant stress and increased red cell turnover, and facilitate the development of acquired immunity, similar to a mechanism suggested for sickle cell trait.

## Introduction

Haptoglobin (Hp) is an acute phase protein that binds haemoglobin released during the intravascular lysis of erythrocytes. Cell-free plasma haemoglobin is a potent pro-oxidant and haptoglobin is thought to be important in removing it from circulation and recycling the iron component for erythropoeisis via the reticulo-endothelial system and binding of CD163 on circulating monocytes and macrophages [1].

Haptoglobin phenotypes and genotypes have been variously reported to be associated with a range of disease conditions, reviewed by McDermid et al [2], including viral load and mortality in HIV [3], [4], [5], Chaga's disease [6] diabetes-associated cardiovascular disease [7], [8], diabetes [9], gestational diabetes [10] and in some studies, susceptibility to malaria [4], [11], [12], [13], although other studies have not reported such an association [14], [15].

In humans, Hp is polymorphic with two co-dominant alleles, Hp1 and Hp2 encoded by a single gene on chromosome 16, resulting in three phenotypes Hp11, Hp12 and Hp22. Unlike Hp11 and Hp12, Hp22 exists as large circular polymers, whilst Hp12 phenotypes form a variety of polymer formations. Hp22 is reported to have decreased binding affinity for free haemoglobin [16], [17] and has been associated with markers of oxidant stress [7], [18], [19] and iron delocalisation [20]. Several studies have demonstrated plasma Hp concentrations to be affected by Hp phenotype/genotype [18], [21], [22], with circulating concentrations observed in the order of Hp11>Hp12>Hp22. Hp is also thought to affect immune regulation. The different phenotypes have been associated with the Th1/Th2 balance of cytokine response [23], [24], possibly through cross-linking of CD163 on monocytes and macrophages [1], [24], [25] and with lymphocyte subset and levels of CD22 expression [26]. Additionally, Hp is reported to bind to the integrin receptor CD11b/CD18, primarily expressed on monocytes and involved in inflammation, phagocytosis and cytoadherence [27]. In relation to malaria, Hp has been demonstrated to have direct toxic effects on malarial parasites *in vitro* [28] and Hp knockout mice have been reported to have higher parasite densities than wild type mice [29].

A fourth phenotype, Hp0 (i.e. no detectable circulating haptoglobin) has been reported in various African populations. In cross-sectional studies, the occurrence of ahaptoglobinaemia has been associated with malaria endemicity [30], [31], [32] and in some studies with parasite density [22]. Furthermore, haptoglobin concentrations have been shown to increase after malaria treatment [33]. It has been hypothesised that malaria, or other causes of haemolysis, lead to depletion of circulating Hp as it is saturated and taken up by monocytes and macrophages–via CD163. Hence the main cause of apparent ahaptoglobinaemia may be environmental. However, additional factors appear to act to determine Hp levels as the prevalence of ahaptoglobinaemia was not increased in children under 5 yrs of age with higher parasite densities compared to adults with low parasite densities [32], [33]. More recently, haptoglobin concentrations have been associated with parasite density, interacting with age, plus haptoglobin genotype in Gabonese children [22] and alpha thalassaemia genotype in Papua New Guinean children [21].

In the Japanese a genetic basis of ahaptoglobinaemia has been identified as the homozygous inheritance of a deletion spanning the entire Hp gene (Hp-del) [34]. So far this allele has not been detected in African populations [35]. However, two single nucleotide polymorphisms (SNPs) in the promoter region of the Hp gene, A-61C (rs5471) and C-101G (rs5470) have been associated with ahaptoglobinaemia and hypohaptoglobinaemia, respectively in Ghanaian samples, free from malarial parasitaemia [35]. We have investigated the prevalence of these SNPs and of Hp-del in a population of Gambian children, and their association with Hp genotype, Hp concentration and risk of malaria.

## Methods

### Patients and methods

Ethical permission for the study was granted by the Gambian Government and Medical Research Council Ethics Committee, and Gambian National DNA Collection Guidelines were followed regarding the handling of genetic material and information. Parental written informed consent was obtained for all study participants.

The main study described consists of a longitudinal study with active surveillance for malaria and febrile episodes across the malaria transmission season in 2003, in children aged 10–72 months. This was conducted in eight rural villages in the West Kiang District of The Gambia, including the village of Keneba in which the Medical Research Council (MRC) clinic and laboratories are situated. Additionally two cross-sectional surveys of malarial parasitaemia prevalence were conducted, before (N = 873) and after (N = 801) the malaria transmission season in July and December 2003.

At the cross-sectional surveys, children found to have anaemia (Hb<80 g/l) were treated with iron sulphate supplements (30 mg/day for 30 days), whilst children found to have malaria parasites of any density were treated with chloroquine and suphadoxine-pyrimethamine as per Gambian government treatment guidelines. The definition of malaria used for the cross-sectional survey was slide-confirmed parasitaemia of any density.

The longitudinal surveillance study enrolled 889 children. Children were not eligible if they were known to have sickle cell disease or were found to have protein-energy malnutrition as determined by weight-for-height z-score of less than minus three (such children were referred to the Keneba supplement centre for treatment). DNA samples were available from 735 of the enrolled children. One child later found to be homozygous for HbS was excluded from the final analysis. Complete genetic and clinical follow up data were available for 599 children, whilst 1 further child was excluded from the final analysis as lost to follow up after only 1 week. Village assistants measured children's temperatures twice weekly and children with recorded temperature (>37.5°C) were seen by the study clinician at a twice weekly mobile clinic in each village. In addition, mothers were encouraged to bring their children to the mobile clinic if they felt their children had fever or was unwell. Those children with an increased temperature (>37.5°C axilla) or whose mothers reported a history of fever in the previous 48 hours were tested for malaria infection using a dipstick (ICT malaria Pf/Pv, AMRAD-ICT, Brookvale, Australia). Children with positive dipstick results had a 4 ml blood sample collected and parasitaemia confirmed by microscopy (12 out of the 271 positive dipstick tests were negative by slide result). Three different definitions were used to define malarial episodes: –(i) recorded temperature >37.5°C within the previous 3 days, plus a positive malarial dipstick, confirmed by microscopy to have asexual stage parasites >500/ul of blood (N = 255); (ii) as for (i) but including all those that were dipstick positive (N = 271) (iii) dipstick positive with a recorded temperature >37.5°C within the previous 3 days, plus clinical judgement of malaria by the study clinician, initially blinded to the dipstick result (N = 167). Malaria as a cause of subsequent febrile episodes was determined based on clinical judgement and few were confirmed by dipstick or microscopy. All children seen by the study clinician at these clinics received treatment as appropriate, either in the field or at MRC Keneba clinic and free of charge. Thus all first malaria episodes were detected and confirmed by microscopy.

### Laboratory procedures and statistical analysis

Blood films were stained with Field's and Giemsa, and examined for malaria parasites according to standard methods. Frozen plasma samples were analysed at the MRC Human Nutrition Research Laboratories Cambridge, UK. Plasma haptoglobin and CRP concentrations were measured by immunoturbidimetry, Tina-quant on a Hitachi 912 Bioanalyser (Roche Systems, Basel, Switzerland). DNA was extracted from peripheral blood leukocytes using standard methods [36] and quantified using the PicoGreen assay with measurement of flouresence by the TECAN SPECTRAfluor Plus fluorimeter. Frozen aliquots of DNA were shipped to The Wellcome Centre for Human Genetics, Oxford, UK, where all genotyping was conducted using genomic DNA. Haptoglobin was genotyped by allele-specific PCR adapted from a method published by Koch *et al.* [37]. This method determines the Hp1 and Hp2 alleles but does not distinguish between the “F” and “S” subtypes of the Hp1 and Hp2 alleles and therefore avoids potential misclassification based on the presence of different combinations of these sub-types [38]. The presence of the previously reported haptoglobin deletion was assessed using the method of Koda *et al.* [39] who kindly provided control DNA samples known to be heterozygous and homozygous for this deletion. PCR products were resolved in 1% agarose gels, stained with ethidium bromide and visualised under UV light. Details are provided in the supplementary material, Tables S1 and S2.

Sequenom^®^ MassARRAY^®^ (Sequenom^®^, Hamburg Germany) was used to genotype the following SNPs according to manufacturer's instructions: Haptoglobin promoter SNP's A-61C (rs5471) and C-101G (rs5470), HbS (rs334: haemoglobin–sickle), plus the A-376G (rs1050829) SNP in G6PD (glucose-6-phosphate dehydrogenase) resulting in mild enzyme deficiency, type A phenotype [40]. We did not type the G-202A SNP, which when inherited with -376G allele results in a more severe phenotype, A- [40] as we have previously found the prevalence to be very low in this population (Atkinson-SH, unpublished data). Primer details are given in the supplementary material, Table S3.

Due to the unexpected lack of homozygotes for the -61C allele of the Hp promoter SNP (see results section below), we generated two control plasmids for the A-61C SNP, one containing the A allele and the other the C allele. Briefly, gDNA was PCR amplified using the first-round primers in Table S3 as per the Sequenom standard protocol from a pool of gDNA samples identified as heterozygote during genotyping. The PCR product obtained was cloned into the pGEM-T vector (Promega, Southampton, UK) according to the manufacturer's protocol. Colonies were screened by PCR plus restriction enzyme digestion with BpmI (NEB, Hitchin, UK) (Figure S1). Positive colonies for each allele were grown and plasmid DNA prepared. The plasmids were then genotyped individually or as a mixture using the Sequenom^®^ MassARRAY^®^. This confirmed that the assay was able to correctly identify the AA, AC and CC genotypes (data not shown).

Haplotypes for the two Hp promoter SNPs and the haptoglobin gene alleles–Hp1&Hp2–were constructed using SNPHAP 1.1 [41].

Analyses were conducted using STATA version 9 (Stata, StataCorp LP, Texas, USA). Associations between genotypes were tested using Pearson's Chi-square test and binomial regression, which was also used to determine whether alleles were geographically clustered.

Analysis of associations between Hp haplotypes, HbS and G6PD genotypes and the prevalence of malarial parasitaemia (any density) at the cross sectional surveys, was performed using logistic regression controlling for age, sex and village. Analysis of associations between Hp haplotypes, HbS and G6PD genotypes and risk of at least one malarial episode, assessed in the longitudinal cohort, was performed by both Cox-regression–time to first malarial event, and logistic regression, whereby children were classified as having had one or more malarial episodes or none. Children who did not have data for all of the genes tested were not included in this analysis. The mean follow up time of the 598 children included in the analysis was 99 days (SD 9.6) whilst the minimum and maximum follow up times were 21 and 102 days. No material differences were observed between models using Cox-regression time to first event or logistic regression. Hence, here we report results of the logistic regression models. Age, sex and village were included in all models a-priori. Hp haplotypes were fitted as a dose effect, coded by the number of copies (0, 1 or 2). Thereafter if a haplotype was found to be associated, we attempted to test for a dominance effect (i.e. to test if the effect per copy was linear or differed in the heterozygote or homozygote condition). However, the small numbers of individuals with more than 1 copy of the relevant haplotype resulted in break-down of the assumptions of the model and the results were uninterpretable. Children who were found to be HbSS were excluded from analyses and therefore sickle genotype was fitted as a simple binary variable, whilst G6PD A376-G was fitted taking into account the phenotypic affects of sex-linkage by coding males with one copy of the mutant 376-G allele (G6PD AZ) and females with two copies of the 376G allele (G6PD AA), similarly.

Malarial parasitaemia at the pre-season cross-sectional survey was assessed but not found to be associated with risk of subsequent malaria during surveillance.

Comparison of concentrations of plasma haptoglobin (log-transformed) measured at malaria episodes and at convalescence was assessed using a paired student's t-test with levels less than the minimum detectable level (MDL, 20 ng/dl) assigned a value of 10 ng/dl. However, a considerable proportion of samples, especially at convalescence were below the MDL. Therefore further analyses were conducted of the proportions of samples above and below the MDL–at malaria and convalescence–and associations with Hp haplotypes and genotypes using multiple logistic regression.

## Results

Genotypes were available for between 665 (haptoglobin) and 755 (G6PD–G376-A) children for each of the six genes tested. The overall prevalence of the genotypes for each gene did not vary between the total number and the 598 children included in the final analysis of the longitudinal study.

The prevalence of the different genotypes for the 598 children included in the final analysis of the longitudinal study is presented in **Table 1**. Similar to that previously reported in a smaller populations of Ghanaians [35], [42] we did not detect the presence of the Hp-del allele, of which the homozygous inheritance has been proven to be the major cause of ahaptoglobinaemia in Asian populations [34], [39]. Additionally, we did not detect the presence of any homozygotes for the haptoglobin promoter A-61C allele. The distribution of genotypes for this promoter SNP and also, haptoglobin itself, were not in Hardy Weinberg equilibrium.

**Table 1:** Prevalence of tested genotypes in individuals included in malarial surveillance analysis (N = 598)

Gene	genotypes					
Hp	Hp11	21.1%	Hp12	57.0%	Hp22	21.9%
A-61→C	AA	73.4%	AC	26.6%	CC	0%
C-101→G	CC	74.4%	CG	24.1%	GG	1.5%
HbS	HbAA	83.4%	HbAS	16.6%	HbSS	0%
G6PD^376^	BB	56.9%	BA	22.9%	AA or AZ	20.2%
Hp ^del^ ^*^	Hp^WT/WT^	100%	Hp^WT/del^	0%	Hp^del/del^	0%

* Hp del allele tested for in all samples for which we had not been able to determine a haptoglobin genotype, due to repeated failure of one or both of the primer reactions and also in all homozygotes.

Haplotypes were constructed for the haptoglobin gene and the two haptoglobin promoter SNPs. Four common and two uncommon haplotypes were detected (**Table 2**). These indicated that, as previously reported [35] the -61C allele was highly associated with the Hp2 allele (haplotype D: 101C, -61C, Hp2–13%), whilst the -101G allele was associated with the Hp1 allele (haplotype E: -101G, 61A, Hp1–13%) (**Table 2**). No haplotypes containing the mutant alleles for both of the promoter SNPs were detected. However, there were a small number of combined heterozygotes for the promoter SNPs 4% (24/598) with co-inheritance of the two common mutant haplotypes–D&E. The association between the -61C allele and the Hp2 allele, combined with the observed lack of homozygotes for -61C likely explains why these genes were not observed in Hardy Weinberg equilibrium.

**Table 2:** Prevalence of haplotypes constructed from haptoglobin alleles and haptoglobin promoter SNPs.

					Distribution of haplotype dose (N = 598)		
Haplotype	C-101G	A-61C	Hp	Haplotype frequency (n = 2*598)	0	1	2
A	C	A	1	36%	226 (38)	313(52)	59 (10)
B	C	A	2	37%	232 (39)	289 (48)	77 (13)
C	C	C	1	0.5%	594 (99)	4 (1)	0 (0)
**D**	**C**	**C**	**2**	**13%**	**442 (74)**	**156 (26)**	**0 (0)**
**E**	**G**	**A**	**1**	**13%**	**447 (75)**	**144 (24)**	**7 (1)**
F	G	A	2	0.5%	594 (99)	4 (1)	0 (0)

* Shaded blocks indicate possession of the mutant allele for the gene indicated.

Multiple binomial regression analysis of the Hp 2 allele revealed geographic clustering in some villages (2/9) plus independent associations with the number of -61C and -101G alleles (coefficient 2.65, OR = 14.2 [95% CI 5.1–39.4], p<0.001&−1.32, OR = 0.27 [95% CI 0.2–0.4], p<0.001, respectively) and also G6PD deficiency, whereby male hemizygotes (AZ) and female homozygotes (AA) were less likely to have the Hp2 allele (coefficient -0.83, OR = 0.44 [95% CI 0.3–0.7], p = 0.002). No associations between the haptoglobin promoter SNPs and HbS and G6PD deficiency alleles were detected.

### The A-61C SNP is associated with protection from malaria

#### Cross-sectional surveys of malarial parasitaemia

The prevalence of malarial parasitaemia was 8.6% at both the pre-season (50/585) and post-season surveys (49/568), limited to those with Hp haplotype data available. There were no differences in the prevalence of malaria between individuals with and without complete genotype data available (data not shown). At the pre-season cross-sectional survey, in multiple logistic regression, controlling for age (grouped in years), sex and village, the prevalence of malarial parasitaemia (any density) was significantly decreased in individuals with 1 copy of haplotype “D” (-101C, -61C, Hp2; OR = 0.30 [95% CI 0.12–0.80], p = 0.014). As no homozygotes for -61C were detected there were no individuals with two copies of this haplotype. No other genotype associations were detected at this time point. At the post-season survey there was no evidence for an association with malaria for any of the haplotypes or with HbAS or G6PD genotypes.

#### Longitudinal study of malaria risk

Of children enrolled in the longitudinal study, 598 children had complete genotype data, of which 255 were detected as having at least one malarial episode as described previously, with a parasite density greater than 500 asexual parasites/ul. Of these children, only three were recorded as having a subsequent malarial episode–as determined by clinical judgement by the study clinician. Sex and age groups were evenly distributed amongst the 598 children included in the final analysis.

**Figure 1:**
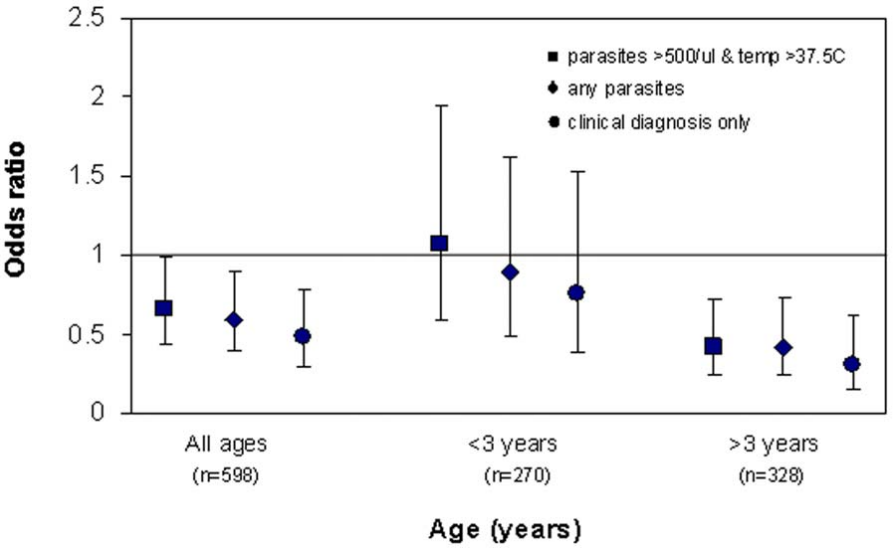
Likelihood of one or more malarial episodes associated with presence vs absence of haplotype D (-101C, -61C, Hp2) in all ages combined and<and >3 years of age (controlling for number of copies of haplotype E, sex, village and HbAS).

Children with one copy of Hp haplotype “D” (-101C, -61C, Hp2) had a significantly decreased risk of having a malarial episode (definition (i) >500 parasites/ul and history of or current temp >37.5°C) (OR = 0.66, p = 0.043, **Table 3**), controlling for age (grouped in years), sex, village and HbAS, the latter of which was also protective (**Table 3**). The protective effect of the “D” Hp haplotype appeared to be limited to older children (**Figure 1**) with no apparent effect in children aged less than three years but a significant effect in children aged 3 or more years (OR = 0.42 [95% CI 0.24–0.73], p = 0.002). Additionally, the interaction between the presence/absence of the “D” haplotype and age, treated as a binary variable as more or less than 3 years, was statistically significant (lr test, p = 0.014). There was no difference in the prevalence of the “D” Hp haplotype between the different age groups (26% vs 25%).

**Table 3:** Genotypes and risk of one or more malarial episodes over the malarial transmission season 2003–children aged 10–72 months.

Risk of malarial episode (>500 parasites/ul)(N = 255/598)*	OR	95% CI		P value
**Variable**				
1 vs zero copies of Haplotype “D” (-101C, -61C, Hp2)	**0.66**	**0.439**	**0.986**	**0.043**
Per copy of Hp haplotype “E” vs zero copies (-101G, 61A, Hp1)	1.23	0.853	1.781	0.26
HbAS vs HbAA	0.62	0.381	1.008	0.054
**Risk of MALARIAL EPISODE (any parasites) N = 271/598)** **				
1 vs zero copies of Haplotype “D” (-101C, -61C, Hp2)	**0.60**	**0.397**	**0.893**	**0.012**
Per copy of Hp haplotype “E” vs zero copies (-101G, 61A, Hp1)	**1.50**	**1.030**	**2.167**	**0.031**
HbAS vs HbAA	0.63	0.382	1.003	0.059
**Risk of CLINICAL DIAGNOSIS (N = 167/598)** ***				
1 vs zero copies of Haplotype “D” (-101C, -61C, Hp2)	**0.48**	**0.296**	**0.777**	**0.003**
Per copy of Hp haplotype “E” vs zero copies (-101G, 61A, Hp1)	1.22	0.809	1.790	0.32
HbAS vs HbAA	**0.51**	**0.281**	**0.906**	**0.019**

* Model LR chi2(15) = 54.68, Prob >chi2 = <0.0001, Pseudo R2 = 0.0671.

** Model LR chi2(15) = 66.71, Prob >chi2<0.0001, Pseudo R2 = 0.0811.

*** Model LR chi2(15) = 60.81, Prob >chi2<0.0001, Pseudo R2 = 0.0859.

Other variables included in all models included age (ordered categorical grouped in years, <2 as reference group then <3, <4, <5&<6); sex (M vs F) village of residence (8 villages as an ordered categorical variable).

Clinical diagnosis was based on clinical judgement at presentation by study clinician blinded to positive malarial dipstick and blood film results.

**Figure 2:**
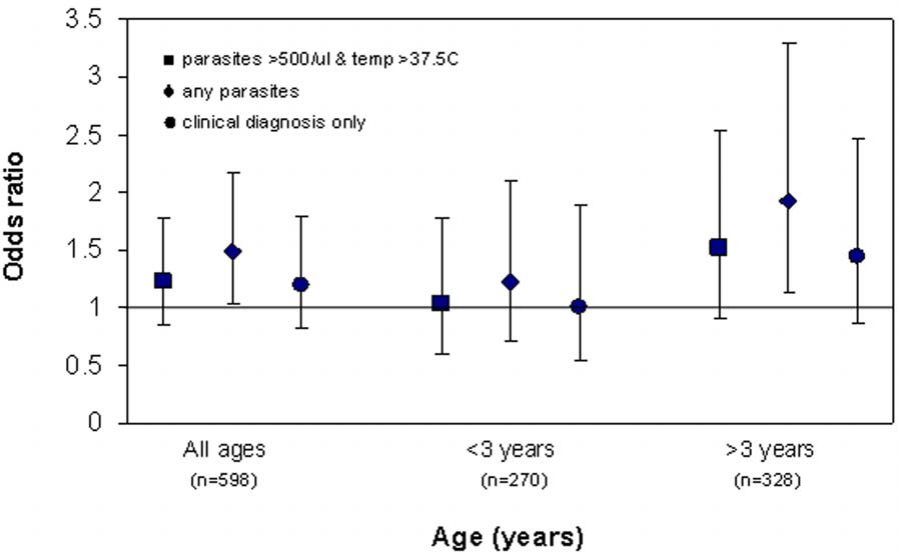
Likelihood of one or more malarial episodes per copy of haplotype E (-101G, -61A, Hp1) in all ages combined and<and >3 years of age (controlling for presence/absence of haplotype D, sex, village and HbAS).

If the definition of a malarial episode was relaxed to include all those that were dipstick positive (N = 271/598), results from the multivariate analysis were similar for haplotype “D” (OR = 0.61, p = 0.012, for all ages. **Table 3**), with the addition that haplotype “E” (-101G, 61A, Hp1) was found to be significantly associated with an increased risk of malarial infection (OR = 1.50, p = 0.031, for all ages) (**Table 3**). Both of these effects were limited to children aged 3 years or more (“D”: OR = 0.42, [95% CI 0.24–0.74] p = 0.002; no of copies of “E”: OR = 1.94 [95% CI 1.15–3.27] p = 0.013), (**Figure 2**). Finally, a further definition of malaria was tested, blinded clinical diagnosis of malaria, based on the study clinician's judgement on examination, plus a positive dipstick result (N = 167/598). Using this definition there was no apparent effect of the “E” haplotype, either when all age groups were included or when limited to older children. There remained the protective effects of haplotype “D” (OR = 0.48, p = 0.003, all age groups) (**Table 3**), whilst the effects of “D” and also of HbAS were both limited to the older children (“D”: OR = 0.3 [95% CI 0.15–0.60], p = 0.001; HbAS: OR = 0.30[95% CI 0.8–2.4], p = 0.008) (**Figure 1**&**2**).

### Haptoglobin genotype and SNP promoters are associated with plasma haptoglobin concentrations

Haptoglobin concentrations were analysed at malaria (n = 257, first detection of a confirmed malarial episode, parasite density >500/ul plus temperature >37.5°C) and convalescence (n = 235, 14 days later&no detectable parasites) in individuals with Hp haplotype data available. Alternatively, samples with concentrations less than this detection limit were coded as being “hypo-haptoglobinaemic”. Contrary to previous reports, the geometric mean concentration of haptoglobin was increased at malaria compared to convalescence (56.13 mg/dl vs 27.50 mg/dl, paired t-test, p<0.0001, n = 231). Additionally, the prevalence of hypo-haptoglobinaemia (defined as values below the assay sensitivity) was significantly increased at convalescence compared to malarial episodes 54.9% vs 30.0%, (two-sample test of proportions, p<0.001). As expected, the frequencies of haplotypes D and E differed between the group who had a malarial episode and were thus included in analyses of Hp concentrations and the group in analysis of malarial risk. Haplotype prevalence was 10.3% and 14.2% for D and E haplotypes in analyses of haptoglobin concentration compared to 13% for both haplotypes in analysis of malaria susceptibility.

Interestingly, different haplotypes were associated with the risk of hypo-haptoglobinaemia at malaria and at convalescence. During malaria, the only haplotype to be associated was haplotype “A” (-101C, -61A, Hp1), associated with a decreased risk (OR = 0.73, p = 0.002) (**Table 4**). Other factors independently associated with individuals being “hypo-haptoglobinaemic” included age, CRP concentration and haemoglobin concentrations. At convalescence the possession of the D and E haplotypes with the mutant alleles of the A-61C and C-101G promoter SNP's were associated with an increased (OR = 2.3, p = 0.023) and decreased risk (OR = 0.41, p = 0.002) of hypohaptoglobinaemia (**Table 4**), whilst age and haemoglobin concentration were no longer associated.

**Table 4:** Multiple binary regression analysis of haplotype effects on risk of undetectable plasma haptoglobin (<20 ng/dl) measured during a malarial episode (>500 parasites/ul) and 14 days later at convalescence and confirmed parasite clearance.

AT MALARIA (N = 257)*				
Variable	Coefficient	95% CI		P value
Per copy of Hp haplotype “A” vs zero copies (101C, 61A, Hp1)	−0.853	−1.399	−0.307	0.002
Age grouped in years	0.701	0.423	0.989	<0.001
Haemoglobin g/l	−0.050	−0.074	−0.026	<0.001
CRP mg/l (log10)	0.703	0.285	1.121	0.001
**AT CONVALESENCE (N = 235)** **				
1 vs 0 copies of Hp haplotype “D” (101C, -61C, Hp2)	0.839	0.118	1.561	0.023
Per copy of Hp haplotype “E” vs zero copies (-101G, 61A, Hp1)	−0.899	−1.470	−0.328	0.002
CRP mg/l (log10)	0.381	0.072	0.690	0.016

* LR chi2 (7) = 85.57, p<0.0001, Adj R-squared = 0.2622

** LR chi2 (6) = 26.22, p = 0.0001. Adj R-squared = 0.0795

There was no evidence of an association of HbAS or with G6PD deficiency with hypo-haptoglobinaemia at either time point.

## Discussion

### Haptoglobin and malaria association studies

We report for the first time, that the A-61C Hp promoter SNP is associated with protection from clinical malaria and that this effect is limited to older children, in this analysis, children between 36 and 72 months. We also confirm the previous report that this SNP is highly associated with the Hp2 allele [35]. We postulate that this association with the Hp2 allele might underlie the previous reports of increased malarial susceptibility associated with Hp11 phenotype [12], [13], [43]. Interestingly, in two case control studies in Sudan and Southern Ghana the prevalence of the Hp12 phenotype as well as Hp22 was also much greater in the controls compared to the cases [12], [43] thereby suggesting protection associated with the Hp2 allele. Similarly, the prevalence of placental parasitaemia was also reduced in both Hp12 and Hp22 phenotypes compared to Hp11 [13]. Moreover, differences in population prevalence of the A-61C and potentially also, the C-101G SNP could conceivably explain contradictory results as observed in a Northern Ghanaian population [15]. The lack of an association in a previous Gambian case-control study could potentially be due to an underestimate of the Hp2 allele due to the use of the genotyping method by Yano et al [44], which misclassifies the Hp2ff and Hp2ss subtypes as Hp1 as the African prevalence of these Hp2 subtypes, compared to Hp2fs is not currently known. The studies of malarial susceptibility associated with Hp11, and therefore a protective effect of Hp2, have been criticised for employing Hp phenotyping, in which it is not possible to determine the phenotype in those with low levels of Hp (Hp0), which our results suggests is associated with the Hp22 genotype, especially during malarial infection. However, in one study, the prevalence of Hp0 was reported not to differ between cases and controls, although absolute numbers were not reported [43] and in another, no Hp0 phenotypes were found [13]. Finally, in support of our finding, a recent analysis of a cohort of Kenyan children with 558 child years of active surveillance for malarial incidence, demonstrated a significant protective effect of the Hp22 genotype [45]. Furthermore, the simultaneous detection in our study of protection in individuals heterozygous for HbS, independent of an effect of the A-61C base substitution, and of a similar magnitude, adds weight to this association.

### Potential mechanisms for a protective effect of the A-61C SNP from malaria infection

The A-61C SNP has been demonstrated to cause decreased promoter activity in human HepG2 cells and decreased responsiveness to IL-6 [35], [46] and has been associated with the Hp0 phenotype in non-malarial Ghanaian samples [35]. In our population of Gambian children, the association between the -61C allele and undetectable Hp concentration was only evident at convalescence and not during malarial episodes, when IL-6 is likely increased. However, during malaria episodes, two separate mechanisms affect Hp concentrations, levels of expression and also rate of clearance, affected by Hp phenotype, either due to differences in affinity of Hp to CD163 [1] or rates of receptor mediated endocytosis [8]. At convalescence, assuming the absence of malaria-induced haemolysis, only levels of expression affect Hp concentration. Somewhat surprisingly, Hp concentration during malarial episodes was not found to be associated with parasite density. However, assuming such a relationship between parasite density and haptoglobin production exists, the timing of peak parasitaemia, haemolysis and acute-phase stimulation of Hp synthesis (and its subsequent sequestration by macrophages) are unlikely to precisely coincide, making its detection in naturalistic studies of malarial episodes difficult to detect. In our study, in cases of confirmed symptomatic malaria (current or record of temperature >37.5°C, and parasite density >500/ul) age was negatively correlated with haptoglobin concentration–perhaps suggesting a decreased acute phase/inflammatory reaction in older children. Our observation that the protection afforded by the -61C allele was limited to older children also suggests that this effect may be mediated via immune mechanisms. The protective effect of HbAS has been documented in some populations to increase with age [47] thus suggesting an immune as well as an innate component to protection. Data from two other studies have suggested that this may be associated with an increased rate of development of immune mechanisms and antibody repertoire [48], [49]. Additionally, protection does not appear to extend to mild or non-symptomatic malaria [50]. A universal mechanism of innate protection has been proposed for G6PD deficiency, HbAS and HbC in which there is an increased rate of phagocytosis of ring-stage parasitised mutant red cells, similar to the mechanism of removal of senescent or damaged red cells [51]. The link between an innate mechanism such as suggested above and immune effects could be via the maintenance of low-grade, chronic infections in infancy and thus an increased rate of development of immune mechanisms and antibody repertoire, resulting in protection from severe/symptomatic malaria in older children HbAS [47], [52]. Therefore a similar mechanism to this could be responsible for the effect of the -61C allele, whereby decreased Hp concentrations causes increased oxidant stress, increased levels of senescent/damaged red cells and therefore increased red cell turnover and perhaps increased phagocytosis of infected red cells. Alternatively, or in addition, the reduced synthesis of haptoglobin during malarial infections could have effects on the inflammatory response and immune functions via reduced signalling through CD163, CD22 or MAC1 (CD11b/CD18) receptors [23], [24], [25] and modulation of the immune response [18], [24].

The -61C allele has been found to be the cause of a “modified” phenotype of Hp as assessed in gel electrophoresis as Hp12_MOD_ [53] thought to be caused by lower expression of the Hp2 polypeptide compared to the Hp1 polypeptide and the formation of different polymeric structures. When tested, our observation did not appear to be due to this phenotype (data not shown). However both Hp12 and Hp22 phenotypes can form a wide variety of polymer formations [17], [53], possibly dependent on levels of haemolysis and which may differ in their functional capacities, as documented for haemoglobin binding capacity, [16], [17]. It remains to be seen if the -61C allele is associated with characteristic polymer formation in the Hp12 and Hp22 phenotypes, perhaps through an association with particular Hp2 and Hp1 “f” and “s” subtypes.

It is not clear why, if there are significant benefits of the heterozygous state, we did not observe any homozygotes for the base substitution. In order to rule out methodological error, we retested the ability of the Sequenom method to detect the -61CC genotype using cloned -61C or -61A DNA. In all of the samples tested, the results of the Sequenome method matched those from restriction digests, suggesting that the lack of detection of -61CC homozygotes was not due to methodological error (data not shown). Based on the current results, Hardy Weinberg would predict 12 individuals homozygote for -61C out of the 750 tested, suggesting a balanced polymorphism similar to HbS in which the homozygous inheritance of the -61CC is potentially lethal. In support of this supposition we have not detected any -61CC homozygotes in a large cohort of Tanzanian women and in another cohort of Gambian children (unpublished data). We plan to test a set of umbilical samples to determine if this genotype can be detected in a birth cohort. However, one female homozygote for -61CC out of 17 individuals found to be ahaptoglobinaemic has been reported from 123 tested [35]. No information on age or other clinical indices was given, nor were overall genotype prevelences for this and other SNPs reported, although the allele frequency of -61C was exactly the same as in our study at 12.6%.

In conclusion, we report an age-dependent protective effect of the -61C allele-associated Hp haplotype against symptomatic malaria in Gambian children. We suggest that the mechanism of protection may be similar to that recently proposed for sickle cell trait and are currently investigating if this haplotype is associated with increased oxidant stress, as a critical component of the proposed mechanism of action.

## Supporting Information

Table S1 Haptoglobin Hp1/Hp2-PCR conditions and primer sequences-method adapted from Koch et al. 2003
(0.02 MB XLS)

Table S2 Haptoglobin deletion assay-PCR conditions and primer sequences-method adapted from Koda et al. 2000
(0.02 MB XLS)

Table S3 First Round and Extension Primers for G6PD, HbS, A-61C and C-101G
(0.02 MB XLS)

Figure S1 Bpm1 restriction digest of plasmid colonies
(0.22 MB TIF)
