# Supplementary figures and images for: Haplotype Association between Haptoglobin (Hp2) and Hp Promoter SNP (A-61C) May Explain Previous Controversy of Haptoglobin and Malaria Protection
Source: PLoS One. 2007 Apr 11;2(4):e362. doi: 10.1371/journal.pone.0000362 (PMC1838521; doi:10.1371/journal.pone.0000362)

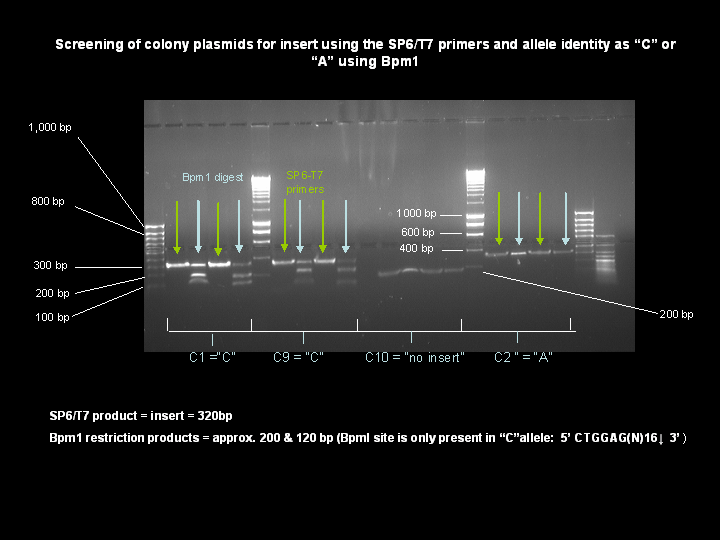

Supplement: Figure S1 — Bpm1 restriction digest of plasmid colonies (0.22 MB TIF) [file pone.0000362.s004.tif]
